# The Impact of Expanding Diabetes Services on the Trend of Glycemic Control in Children and Adolescents with Type 1 Diabetes

**DOI:** 10.1155/2024/5529674

**Published:** 2024-02-09

**Authors:** Nouf Alissa, Shahad Alhumaidi, Sarah Alzaid, Omar Aldibasi, Haifa Alfaraidi, Angham Almutair

**Affiliations:** ^1^Pediatric Department, King Abdulaziz Medical City, Ministry of National Guard Health Affairs, King Abdullah Specialized Children's Hospital, Riyadh, Saudi Arabia; ^2^Ministry of National Guard Health Affairs, King Abdullah International Medical Research Center, Riyadh, Saudi Arabia; ^3^College of Dentistry, Ministry of National Guard Health Affairs, King Saud Bin Abdulaziz University for Health Sciences, Riyadh, Saudi Arabia; ^4^Department of Biostatistics and Bioinformation, Ministry of National Guard Health Affairs, King Abdullah International Medical Research Center, Riyadh, Saudi Arabia; ^5^College of Medicine, Ministry of National Guard Health Affairs, King Saud Bin Abdulaziz University for Health Sciences, Riyadh, Saudi Arabia

## Abstract

**Objectives:**

Our aim is to evaluate the impact of initiating a specialized children's hospital and expanding the diabetes service for children with type 1 diabetes (T1D) on their glycemic control and on acute–diabetes-related complications over a 4-year follow-up period.

**Methods:**

This was a retrospective cohort study that included children aged 1–16 years with T1D, diagnosed for at least 1 year, and treated with multiple daily injections (MDI) or continuous subcutaneous insulin infusion (CSII). The study period extended from January 1, 2016 to December 31, 2019. Outcomes included the trend of glycemic control measured by HgbA1c and acute–diabetes-related complications, such as hypoglycemia, hyperglycemia, and diabetic ketoacidosis (DKA), reflected by the number of emergency room (ER) visits. Additionally, the number of visits per patient per year was captured over the 4-year study period.

**Results:**

Four hundred ninety-nine patients with T1D were included in the study (48.9% female). The mean age was 13.4 years (±2.0) in the CSII group and 12.4 years (±2.2) in the MDI group. Three thousand nine hundred and six visits were reviewed, with 618 in the CSII group and 3,288 in the MDI group. The mean hemoglobin A1c (HgbA1c) for the whole cohort was 10.56% at the start of the study period in 2016 and dropped by 0.67% to a mean of 9.89% in 2019 (*p*-value = 0.025). There was a 0.67% decline in the HgbA1c of the MDI group and a 0.47% decrease in the CSII group (*p*=<0.001). The average number of clinic visits per patient per year increased from 2.6 in 2016 to 2.8 in 2019. ER visits slightly decreased throughout the 4-year period (*p*-value = 0.46).

**Conclusion:**

Increased accessibility of the diabetes care team to children and adolescents with T1D and their families, with more frequent contact with team members, contributes significantly to the improvement of glycemic control.

## 1. Introduction

Type 1 diabetes (T1D) is the most common cause of diabetes mellitus in the pediatric age group, representing one of the most common chronic health conditions in children and adolescents [1]. It is estimated that T1D affects more than 1.2 million children and adolescents globally, with almost 29,000 affected youth in Saudi Arabia. Saudi Arabia is among the top 10 countries with the highest prevalence of T1D in youth under 19 years [2]. Saudi Arabia is the largest country in the Middle East, with a population of over 32 million [3]. The Saudi population is primarily a young population, with a median age of 29 years and almost a quarter of the population under the age of 20 years [3]. Saudi Arabia occupies the ninth position worldwide for annual new T1D cases, with 3,800 new cases diagnosed every year in those aged less than 19 years [2]. It occupies the eighth position worldwide for annual incidence rates, with 31.4 cases per 100,000 youth per year among the 0–14 age group [2]. Local studies have reported a prevalence of T1D in Saudi children and adolescents of 109.5 per 100,000, with rates reaching up to 355 per 100,000 in some regions [4, 5]. Therefore, T1D, a critical healthcare concern in Saudi Arabia, requires allocating resources for proper management. The Saudi government provides free healthcare to its citizens and residents through primary healthcare centers and tertiary hospitals, primarily run by the Ministry of Health and accessible to everyone. The Ministry of Health also funds many specialized diabetes centers distributed throughout the country, where all Saudis and residents are eligible to seek care. The healthcare system also encompasses specific healthcare institutions that provide care, including diabetes care, specifically to eligible populations and their dependents, such as military personnel, National Guard affiliates, or university faculty.

To improve T1D outcomes and reduce complication rates, various strategies and tools have been adapted to optimize glycemic control [6, 7]. The use of newer rapid-acting, long-acting, and ultra-long-acting insulin are readily available in Saudi Arabia. Additionally, advanced diabetes technology, such as continuous subcutaneous insulin infusion (CSII) and continuous glucose monitoring (CGM) devices, helps reduce hemoglobin A1c (HgbA1c) levels as well as hypoglycemia episodes [8–12]. In Saudi Arabia, flash glucose monitoring has been routinely offered to individuals with diabetes through specialized diabetes centers for the last 2–3 years, with the cost covered by the government. While CSII therapy is also available, access to it may be somewhat limited due to its high cost and accessibility may vary between centers.

Diabetes education plays a vital role in effective diabetes management, and the role of the diabetes team should not be overlooked, with evidence supporting a reduction in diabetes ketoacidosis (DKA) admissions with specific diabetes-educator care models [13]. Structured education programs for patients with T1D that focus on self-care can help reduce HgbA1c levels and increase overall knowledge and confidence regarding diabetes care [14]. Furthermore, regular and frequent visits to a multidisciplinary diabetes clinic promote better glycemic control with lower HgbA1c levels [15–17].

A large cohort of children and adolescents with T1D are followed at King Abdulaziz Medical City, in a dedicated children's hospital, King Abdullah Specialized Children Hospital (KASCH), affiliated with the National Guard Health Affairs. The children's hospital started operating in 2015 with a multidisciplinary diabetes team that included seven physicians, two diabetes educators, and a general dietician. In 2016, the diabetes service expanded to include eleven physicians, five diabetes educators, and a dietician specialized in carbohydrate counting. Additionally, there was an increase in the physical space of the clinics, with an increase in the clinic offices available to see patients and their families from 3 clinics per day to 10 clinics per day. This allowed healthcare professionals to spend more time with patients and their caregivers reviewing their diabetes care and emphasizing on education regarding problem-solving skills. Additionally, diabetes technology tools have become more readily available in our service, with an increase in CSII use over recent years. Flash glucose monitoring also became universally available for all patients with T1D in 2019.

Our study aims to evaluate the impact of initiating a children's hospital in 2015, with a subsequent expansion of the diabetes service, on glycemic control and acute–diabetes-related complications. We hypothesized that the growth in service capacity and the increased utilization of diabetes technology would translate into an improvement in glycemic control and a reduction in acute–diabetes-related complications over the 4-year study period.

## 2. Materials and Methods

This retrospective cohort study was conducted at KASCH, one of several hospitals and primary healthcare centers serving individuals affiliated with the National Guard of Saudi Arabia and their dependents. KASCH is a tertiary children's hospital that began operating in 2015, offering specialized care to children and adolescents with chronic medical conditions, including diabetes mellitus. The pediatric diabetes center at KASCH accepts the care of all children of individuals affiliated with the National Guard with no restrictions. Additionally, exceptions are occasionally made for other Saudi children and adolescents with T1D if a treatment request is made, and there are no specific restrictions on them joining the program.

At the pediatric diabetes center at KASCH, CSII therapy is offered to children and adolescents with T1D as per the National Institute of Clinical Excellence (NICE) guidelines, with no constraints based on HgbA1c level or diabetes duration. The NICE guidelines suggest offering CSII therapy to achieve glycemic control in older children and adolescents and as a treatment option for younger children based on the appropriateness and practicality of MDI in this age group [18].

The study population included children and adolescents, ages 1–16 years, with T1D for at least 1 year, followed in KASCH, Riyadh, Saudi Arabia, from January 2016 to December 2019, before the COVID-19 pandemic.

Exclusion criteria included age less than 1-year, other types of diabetes (type 2 diabetes, maturity-onset diabetes of the young, and medication-induced hyperglycemia), presence of any chronic disease other than diabetes, such as celiac disease, hemoglobinopathies and syndromes, and significant psychosocial concerns necessitating home healthcare intervention.

Data collected from electronic medical records included gender, age of the patient, number of visits per year and number of HgbA1c results per year, insulin regimen and types, mode of insulin delivery (CSII or multiple daily injections (MDI)), and year of CSII initiation if applicable. Data on acute–diabetes complications included total emergency room (ER) yearly visits for severe hypoglycemia, severe hyperglycemia without acidosis, and DKA.

Severe hyperglycemia was defined as symptomatic blood glucose readings above 200 mg/dL, necessitating an ER visit but without acidosis. Severe hypoglycemia was defined as an event with severe cognitive impairment requiring assistance from another person to administer carbohydrate and/or glucagon injections and a plasma glucose value below 70 mg/dL [19]. Biochemical criteria for the diagnosis of DKA included hyperglycemia (blood glucose >200 mg/dL), venous pH < 7.3 or serum bicarbonate <15 mmol/L, and ketonemia (blood *ß*-hydroxybutyrate ≥3 mmol/L) or moderate to large ketonuria [20]. HgbA1c is measured at our central lab by high-performance liquid chromatography technique.

Data were analyzed and summarized using descriptive statistics to explore and visualize the trend of HgbA1c and total ER visits over 4 years for the two groups (CSII and MDI). Further, regression analyses were conducted using a fixed effects model for longitudinal data to examine the main effect of time and mode of therapy on HgbA1c. The total ER visits were modeled using the negative binomial regression model with time and mode of therapy as the main effects. The significance level was declared *α* 0.05, and SAS 9.4 was utilized for all statistical analyses.

## 3. Results

**Figure 1:**
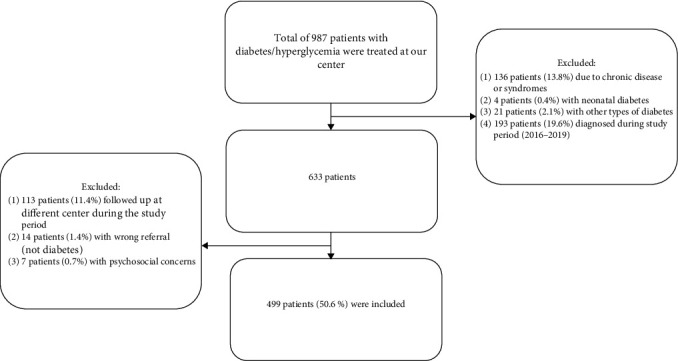
Flow diagram: inclusion process of patients.

From January 2016 to December 2019, 987 patients with diabetes mellitus were treated at KASCH, Saudi Arabia. We excluded 488 patients from the study as they met the exclusion criteria (Figure 1). The final study cohort comprised 499 children and adolescents with T1D, with 255 (51.1%) males and 244 (48.9%) females. At inclusion, all patients were treated with either CSII (*n* = 62, 12.4%) or MDI (*n* = 437, 87.5%) at the first recruitment. The mean age was 13.4 years (±2.0) in the CSII group and 12.4 years (±2.2) in the MDI group. We reviewed 3,906 visits, with 618 in the CSII group and 3,288 in the MDI group.

The mean HgbA1c dropped by 0.67% between 2016 and 2019 for all groups of patients, which is clinically significant. The mean HgbA1c decreased from 10.56% (median 10.30%, range 5.80%−16.00%) in 2016 to a mean HgbA1c of 9.89% (median 9.60%, range 5.30%−16.50%) in the year 2019 (*p*=0.0025; Table 1). The average number of clinic visits per patient per year increased from 2.6 in 2016 to 2.8 (Table 2).

**Figure 2:**
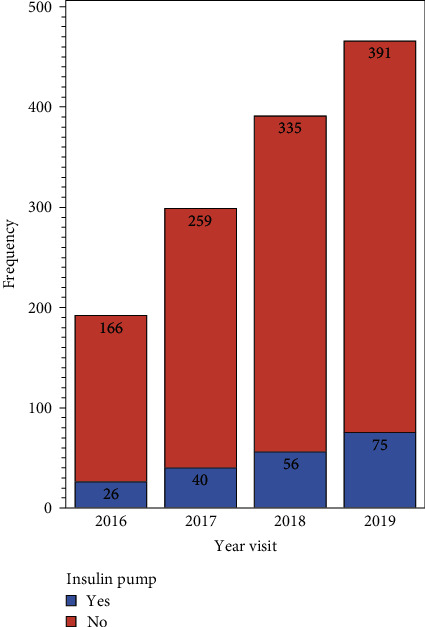
Distribution of pump users per year.

**Figure 3:**
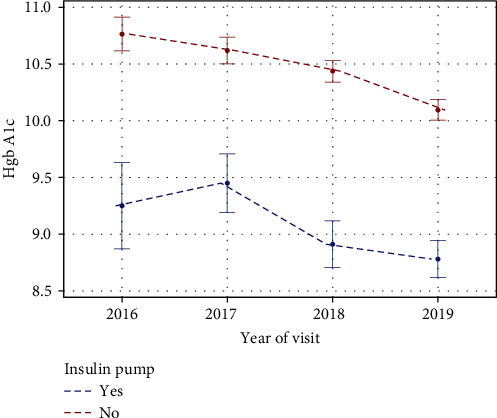
Mean HgbA1c per year for the patient on MDI or CSII (pump).

The use of CSII increased steadily during the 4-year study period, with 26 patients using CSII in 2016 and 75 users in 2019 (Figure 2). Regarding HgbA1c based on the mode of therapy, the mean HgbA1c at the start of the study period was 9.25% (median 8.75%, range 6.70%–14.60%) in the CSII and 10.76% (median 10.65%, range 5.80%–16.00%) in the MDI group. There was a significant decline in the trend of HgbA1c over 4-years in both the MDI and CSII groups (Figure 3), with a 0.47% HgbA1c decline in the CSII group and a 0.67% decrease in the MDI group (*p*-value = <0.001).

**Figure 4:**
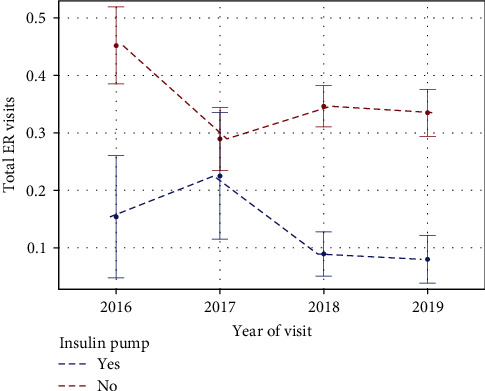
The total ER visits (that include severe hypoglycemia or hyperglycemia or DKA).

The total ER visits for severe hypoglycemia, severe hyperglycemia, or DKA were significantly less in the CSII compared to the MDI (*p*-value <0.0001), with a slight decrease in the trend of total ER visits over 4 years in both groups (*p*-value = 0.46; Figure 4).

## 4. Discussion

Our study aimed to determine the trend of HgbA1c in children and adolescents with T1D followed up at KASCH, a tertiary children's hospital in Riyadh, Saudi Arabia, that was first operated in 2015. We also aimed to determine the impact of the increased implementation of CSII, and the expansion of the diabetes service at the children's hospital with an increase in physicians, diabetes educators, and dieticians specialized in carbohydrate counting on glycemic control and on acute–diabetes complications.

Our results demonstrated an overall decrease in the trend of HgbA1c over the 4-year study, with a reduction of around 0.67%, which is considered clinically significant, as a change of HgbA1c of at least 0.50% or more, when measured in a National Glycohemoglobin Standardization Program (NGSP)–certified laboratory is considered clinically significant based on data from landmark diabetes trials [21].

Many factors may be contributing to these observations. The expansion of the diabetes service at our institution resulted in improved diabetes education, in parallel with an increase in educator to patient ratio from 1 : 450 to 1 : 180. Additionally, there was an increase in the number of patient–physician visits per year, which rose from 2.6 visits per patient per year to around three visits per patient per year at the end of the 4 years. During a typical visit, the patient and their accompanying family member will spend ∼20 min with the physician and another 20 min with a member of the diabetes team (educator or dietician), depending on their need. The average number of visits per patient per year (2.6–2.9 visits per patient per year) is similar to the average number of visits per patient per year reported by Markowitz et al. [3] where the average number of visits per patient over 2 years was 5.8 visits [22]. While the specific attendance rate for the pediatric diabetes clinics is unavailable, overall attendance for all pediatric endocrinology and diabetes clinics remained steady over the 4-year observation period. It ranged from 71.7% to 72.5%. This is in keeping with the attendance rates reported at other diabetes clinics for pediatric and young adult patients, which ranged between 73% and 75% [23, 24].

The degree of contact with the diabetes team was likely even higher, as our data could not capture unscheduled walk-in visits and phone calls with the more readily available diabetes educators. We believe that the increased accessibility of the diabetes care team to children and adolescents with T1D and their families, with more frequent contact with team members, contributed significantly to improving glycemic control. This is supported by evidence demonstrating that quarterly visits with the diabetes team are more effective in improving HgbA1c levels than annual or bi-annual visits [15, 17]. Additionally, we believe that the expansion of our diabetes service led to improved diabetes education and enhancement of diabetes care knowledge in patients and their families, which also contributed to the improvement of HgbA1c. This highlights the importance of continuous diabetes education as an integral component of diabetes care, including education regarding self-management and problem-solving skills. Structured diabetes education programs positively impact glycemic control in individuals with T1D, with a clear association between diabetes knowledge and reduction in HgbA1c [14, 25–28]. Moreover, nurses provide a valuable role in the diabetes team with nurse-led intervention influencing improvements in HgbA1c levels in children and adolescents with T1D [29].

The benefits of improved diabetes education and increased availability and accessibility of the diabetes care team to patients were not only limited to enhanced glycemic control but also extended to acute–diabetes-related complications and ER visits, as there was a trend of reduced ER visits during the 4-year study period, though not statistically significant. This is in keeping with similar observations from both adult and pediatric data, where the utilization of diabetes education programs resulted in fewer ER visits [13, 30]. In youth with T1D, implementing a diabetes educator care model significantly reduced ER visits and DKA admissions over 4 years [13].

Regarding the mode of therapy, glycemic control in the CSII group was markedly better than in the MDI group throughout the observation period. This is unsurprising, as various studies have observed that HgbA1c levels are better in those using CSII than MDI [11, 31–34]. The enhanced glycemic control in the CSII group was maintained throughout the observation period, supporting the notion that the benefit of CSII on glycemic control is preserved on the long term [35–38]. However, even though HgbA1c was lower in the CSII group, the change in HgbA1c was more evident in the MDI group during the 4 years. This can potentially be explained by the fact that individuals who are planning to initiate CSII typically receive intensive diabetes education prior to starting their pump therapy, therefore attenuating the effect of increased subsequent contact with the diabetes team as they are starting with enhanced diabetes care knowledge compared to those treated with MDI. However, continued education, contact, and support from the diabetes team are essential to maintain the improvement in glycemic control in the CSII group, as reflected by the further drop in HgbA1c levels.

We also observed an increase in technology implementation, with an apparent increase in CSII users, from 26 in 2016 to 75 in 2019. We believe this reflects the expansion of our diabetes service, especially in terms of physicians, educators, and dieticians. Unfortunately, due to the retrospective nature of our study, we were unable to determine accurately the effect of CSII use on overall glycemic control. Flash glucose monitoring was only introduced in our institution in 2019, toward the end of our observation period. Therefore, we could not assess the impact of the introduction of flash glucose monitoring on glycemic control.

There has always been a theoretical risk of increased DKA with CSII use, as the insulin pump only administers rapid-acting insulin as basal and bolus doses, and no long-acting insulin is present in the background. Therefore, any interruption in insulin delivery can lead to the rapid development of hyperglycemia, ketosis, and ensuing acidosis. However, in our study, ER visits for hypoglycemia, hyperglycemia, and DKA were significantly lower in the CSII group compared to the MDI group. These results support the safety of insulin pumps, provided users receive adequate diabetes education and training. Our observations are consistent with more recent studies, including large-scale registry data, that have not identified a significant increase in the risk of DKA with CSII use [11, 39–41].

Limitations of our study include its retrospective nature, making clear causation challenging to establish. Although the use of CSII increased during the 4-year study period, we could not directly quantify the impact of this increase on the glycemic control of our cohort. Additionally, we could not assess the effect of flash CGM on glycemic control as it only became available during 2019, the last year of our observation period. Before that, CGM use was limited, and only a few patients with a high risk of developing hypoglycemia were offered CGM. While we did capture the number of physician clinic visits per patient per year, additional information such as time spent with the health care provider during each visit, educator-specific visits, unscheduled walk-in visits, or phone calls to the diabetes team were not captured. These parameters would have provided valuable information to futher quantify the increased education and support provided by our diabetes team.

## 5. Conclusions

The trend of glycemic control in children and adolescents with T1D, followed at our institution, showed a significant improvement over 4 years. We believe this is a reflection of the expansion of our diabetes service over this period, translating into enhanced diabetes education. Our study highlights the importance of diabetes education, patient empowerment, and their crucial impact on glycemic control.

## Figures and Tables

**Table 1:** The trend of HgbA1c over 4 years, 2016–2019.

Year of visit	*N*_MDI_/*N*_CSII_	Overall HgbA1c Mean ± SD	MDI HgbA1c	CSII HgbA1c
2016	166/26	10.56 ± 1.99	10.76 ± 1.92	9.25 ± 1.94
2017	256/40	10.46 ± 1.88	10.62 ± 1.87	9.45 ± 1.64
2018	333/54	10.23 ± 1.78	10.44 ± 1.73	8.91 ± 1.52
2019	381/72	9.89 ± 1.82	10.09 ± 1.81	8.78 ± 1.40

*Abbreviations*. MDI, multiple daily injections; CSII, continuous subcutaneous insulin infusion; SD, standard deviation.

**Table 2:** Average number of clinic visits and HgbA1c results per year.

Year	Number of visits	Number of HgbA1c results	Average visits per patient per year	Number of patient
2016	491	192	2.6	189
2017	845	296	2.7	314
2018	1,185	387	2.9	414
2019	1,385	453	2.8	499

## Data Availability

The datasets used to support the findings of this study are available from the corresponding author upon reasonable request.

## References

[1] Lawrence J. M., Divers J., Isom S. (2021). Trends in prevalence of type 1 and type 2 diabetes in children and adolescents in the US, 2001–2017. *Journal of the American Medical Association (JAMA)*.

[2] International Diabetes Federation (2021). IDF Diabetes Atlas 2021.

[3] Saudi Census Report (2022). Saudi Census 2022.

[4] Al-Ghamdi A. H., Fureeh A. A. (2018). Prevalence and clinical presentation at the onset of type 1 diabetes mellitus among children and adolescents in AL-Baha region, Saudi Arabia. *Journal of Pediatric Endocrinology and Metabolism*.

[5] Al-Herbish A. S., El-Mouzan M. I., Al-Salloum A. A., Al-Qurachi M. M., Al-Omar A. A. (2008). Prevalence of type 1 diabetes mellitus in Saudi Arabian children and adolescents. *Saudi Medical Journal*.

[6] Mochizuki M., Kikuchi T., Urakami T. (2017). Improvement in glycemic control through changes in insulin regimens: findings from a Japanese cohort of children and adolescents with type 1 diabetes. *Pediatric Diabetes*.

[7] Urakami T., Morimoto S., Kubota S., Funaki S., Harada K. (2007). Usefulness of the long-acting insulin analogue glargine in basal-bolus therapy for Japanese children and adolescents with type 1 diabetes mellitus. *Journal of Pediatric Endocrinology and Metabolism*.

[8] Al Shaikh A., Al Zahrani A. M., Qari Y. H. (2020). Quality of life in children with diabetes treated with insulin pump compared with multiple daily injections in tertiary care center. *Clinical Medicine Insights: Endocrinology and Diabetes*.

[9] Benioudakis E., Karlafti E., Kalaitzaki A., Kaiafa G., Savopoulos C., Didangelos T. (2022). Technological developments and quality of life in type 1 diabetes mellitus patients: a review of the modern insulin analogues, continuous glucose monitoring and insulin pump therapy. *Current Diabetes Reviews*.

[10] Bergenstal R. M., Tamborlane W. V., Ahmann A. (2011). Sensor-augmented pump therapy for A1C reduction (STAR 3) study: results from the 6-month continuation phase. *Diabetes Care*.

[11] Karges B., Schwandt A., Heidtmann B. (2017). Association of insulin pump therapy vs insulin injection therapy with severe hypoglycemia, ketoacidosis, and glycemic control among children, adolescents, and young adults with type 1 diabetes. *Journal of the American Medical Association (JAMA)*.

[12] Maiorino M. I., Signoriello S., Maio A. (2020). Effects of continuous glucose monitoring on metrics of glycemic control in diabetes: a systematic review with meta-analysis of randomized controlled trials. *Diabetes Care*.

[13] Deeb A., Yousef H., Abdelrahman L. (2016). Implementation of a diabetes educator care model to reduce paediatric admission for diabetic ketoacidosis. *Journal of Diabetes Research*.

[14] Pacheco A. P. F., de Sande-Lee S., Sandoval R. C. B., Batista S., Marques J. L. B. (2017). Effects of a structured education program on glycemic control in type 1 diabetes. *Archives of Endocrinology and Metabolism*.

[15] Kaufman F. R., Halvorson M., Carpenter S. (1999). Association between diabetes control and visits to a multidisciplinary pediatric diabetes clinic. *Pediatrics*.

[16] Mazarello Paes V., Barrett J. K., Dunger D. B. (2019). Factors predicting poor glycemic control in the first two years of childhood onset type 1 diabetes in a cohort from East London, UK: analyses using mixed effects fractional polynomial models. *Pediatric Diabetes*.

[17] Urbach S. L., LaFranchi S., Lambert L., Lapidus J. A., Daneman D., Becker T. M. (2005). Predictors of glucose control in children and adolescents with type 1 diabetes mellitus. *Pediatric Diabetes*.

[18] Technology appraisal guidance [TA151] (2008). Continuous subcutaneous insulin infusion for the treatment of diabetes mellitus. *National Institute for Health and Care Excellence*.

[19] Abraham M. B., Jones T. W., Naranjo D. (2018). ISPAD Clinical Practice Consensus Guidelines 2018: assessment and management of hypoglycemia in children and adolescents with diabetes. *Pediatric Diabetes*.

[20] Wolfsdorf J. I., Glaser N., Agus M. (2018). ISPAD Clinical Practice Consensus Guidelines 2018: diabetic ketoacidosis and the hyperglycemic hyperosmolar state. *Pediatric Diabetes*.

[21] Little R. R., Rohlfing C. L., Sacks D. B. (2011). Status of hemoglobin A1c measurement and goals for improvement: from chaos to order for improving diabetes care. *Clinical Chemistry*.

[22] Markowitz J. T., Volkening L. K., Laffel L. M. B. (2014). Care utilization in a pediatric diabetes clinic: cancellations, parental attendance, and mental health appointments. *Journal of Pediatrics*.

[23] Fisher E., Lazar L., Shalitin S. (2018). Association between glycemic control and clinic attendance in emerging adults with type 1 diabetes: a tertiary center experience. *Journal of Diabetes Research*.

[24] Nadeem F., Urwin A., Marshall M. (2019). Risk factor control and outpatient attendance in young adults with diabetes. *Acta Diabetologica*.

[25] Dubovi I., Levy S. T., Levy M., Zuckerman Levin N., Dagan E. (2020). Glycemic control in adolescents with type 1 diabetes: are computerized simulations effective learning tools?. *Pediatric Diabetes*.

[26] Hawkes C. P., Willi S. M., Murphy K. M. (2019). A structured 1-year education program for children with newly diagnosed type 1 diabetes improves early glycemic control. *Pediatric Diabetes*.

[27] Martin D., Elie C., Dossier C. (2017). Diabetes knowledge in adolescents with type 1 diabetes and their parents and glycemic control. *Pediatric Diabetes*.

[28] Mauri A., Schmidt S., Sosero V. (2021). A structured therapeutic education program for children and adolescents with type 1 diabetes: an analysis of the efficacy of the “Pediatric education for diabetes” project. *Minerva Pediatrics*.

[29] Bakir E., Sezer T. A. (2023). The efficacy of interventions provided by nurses to improve glycemic control of children with type 1 diabetes: a systematic review. *Journal for Specialists in Pediatric Nursing*.

[30] Gao Y., Xu C., Yang A. (2022). How outpatient diabetes education programs can support local hospitals to reduce emergency department visits for adults with diabetes. *Canadian Journal of Diabetes*.

[31] Kamrath C., Tittel S. R., Kapellen T. M. (2021). Early versus delayed insulin pump therapy in children with newly diagnosed type 1 diabetes: results from the multicentre, prospective diabetes follow-up DPV registry. *The Lancet Child & Adolescent Health*.

[32] Pala L., Dicembrini I., Mannucci E. (2019). Continuous subcutaneous insulin infusion vs modern multiple injection regimens in type 1 diabetes: an updated meta-analysis of randomized clinical trials. *Acta Diabetologica*.

[33] Sherr J. L., Hermann J. M., Campbell F. (2016). Use of insulin pump therapy in children and adolescents with type 1 diabetes and its impact on metabolic control: comparison of results from three large, transatlantic paediatric registries. *Diabetologia*.

[34] Szypowska A., Schwandt A., Svensson J. (2016). Insulin pump therapy in children with type 1 diabetes: analysis of data from the SWEET registry. *Pediatric Diabetes*.

[35] Babiker A., Alammari N., Aljuraisi A. (2022). The effectiveness of insulin pump therapy versus multiple daily injections in children with type 1 diabetes mellitus in a specialized center in Riyadh. *Clinical Medicine Insights: Endocrinology and Diabetes*.

[36] Carlsson B.-M., Attvall S., Clements M. (2013). Insulin pump-long-term effects on glycemic control: an observational study at 10 diabetes clinics in Sweden. *Diabetes Technology & Therapeutics*.

[37] Colino E., Martin-Frias M., Yelmo R., Alvarez M. A., Roldan B., Barrio R. (2016). Impact of insulin pump therapy on long-term glycemic control in a pediatric Spanish cohort. *Diabetes Research and Clinical Practice*.

[38] Johnson S. R., Cooper M. N., Jones T. W., Davis E. A. (2013). Long-term outcome of insulin pump therapy in children with type 1 diabetes assessed in a large population-based case-control study. *Diabetologia*.

[39] Alshami A., Purewal T., Douedi S. (2021). Effect of insulin pump use on diabetic ketoacidosis in type 1 diabetes mellitus: a matched cohort study. *Journal of Clinical Medicine*.

[40] Hoshina S., Andersen G. S., Jørgensen M. E., Ridderstråle M., Vistisen D., Andersen H. U. (2018). Treatment modality-dependent risk of diabetic ketoacidosis in patients with type 1 diabetes: Danish adult diabetes database study. *Diabetes Technology & Therapeutics*.

[41] Karges B., Rosenbauer J., Holterhus P. M. (2015). Hospital admission for diabetic ketoacidosis or severe hypoglycemia in 31,330 young patients with type 1 diabetes. *European Journal of Endocrinology*.

